# Volatile‐Mediated Plant Defense Networks: Field Evidence for Isoprene as a Short‐Distance Immune Signal

**DOI:** 10.1111/pce.70153

**Published:** 2025-09-01

**Authors:** Peiyuan Zhu, Baris Weber, Maaria Rosenkranz, Andrea Polle, Andrea Ghirardo, Jan Muhr, A. Corina Vlot, Jörg‐Peter Schnitzler

**Affiliations:** ^1^ Research Unit Environmental Simulation, Helmholtz Zentrum München Neuherberg Germany; ^2^ Institute of Plant Sciences, Ecology and Conservation Biology University of Regensburg Regensburg Germany; ^3^ Forest Botany and Tree Physiology University of Göttingen Göttingen Germany; ^4^ LARI ‐ Laboratory for Radio‐Isotopes University of Göttingen Göttingen Germany; ^5^ Faculty of Life Sciences: Food, Nutrition and Health, Chair of Crop Plant Genetics University of Bayreuth Kulmbach Germany

**Keywords:** *Arabidopsis thaliana*, *Betula pendula*, isoprene, plant immunity, plant‐to‐plant communication, *Pseudomonas syringae*, terpenoids, volatile organic compounds

## Abstract

Isoprene, the most abundant biogenic hydrocarbon in the atmosphere, is known to protect photosynthesis from abiotic stress and significantly impact atmospheric chemistry. While laboratory studies show that isoprene can enhance plant immunity, its role in plant‐plant communication under natural field conditions remains unclear. In a 2‐year field experiment, we used wild‐type and transgenic silver birch (*Betula pendula*) lines with enhanced isoprene emission levels to examine their impact on neighboring *Arabidopsis thaliana*, including wild‐type and immune signaling mutants (*llp1: legume lectin‐like protein 1; jar1: jasmonate resistant 1*). Receiver plants exposed to higher isoprene levels showed increased resistance to *Pseudomonas syringae*, independent of jasmonate signaling but dependent on LLP1, a protein essential for systemic acquired resistance. Volatile analysis indicated isoprene as an airborne molecule that can also trigger an immune response in neighboring plants along with other terpenoids. Our study using transgenic birches in a complex environment provides new insights into the molecular mechanisms underlying plant volatile perception and expands our understanding of plant chemical communication in terrestrial ecosystems.

## Summary Statement

Transgenic silver birch (*Betula pendula*) with altered isoprene emission capacities enhanced pathogen resistance in neighboring *Arabidopsis thaliana* through a jasmonate‐independent, salicylate‐dependent pathway, demonstrating isoprene's novel role as an airborne immune signal under field conditions.

## 1 Introduction

Plants engage in complex interactions with their environment, employing sophisticated mechanisms to respond to stress and communicate with other organisms. The production and emission of biogenic volatile organic compounds (BVOCs) play a crucial role in plant ecology and defense (Loreto and Schnitzler 2010; Dudareva et al. 2013). BVOCs encompass a diverse range of volatile organic molecules that attract pollinators and seed dispersers, deter herbivores and pathogens, and facilitate plant‐to‐plant communication (Kessler and Baldwin 2001; Peñuelas 2003; Heil and Karban 2010; Howard et al. 2022).

Volatile terpenoids, which contribute the majority of global BVOCs (Acosta Navarro et al. 2014), exist in three primary forms: isoprene (C5), monoterpenes (C10), and sesquiterpenes (C15). Isoprene is the most abundant, contributing approximately 500 Tg year^−1^ of carbon emissions ‐ roughly half of all BVOC‐associated carbon fluxes (Guenther et al. 2012; Yang et al. 2021). By comparison, monoterpenes and sesquiterpenes account for 15% and 3% of BVOC carbon fluxes, respectively. Beyond their quantitative dominance, volatile terpenoids play central roles in plant–environment interactions, functioning in defense, stress tolerance, and communication. (Gershenzon and Dudareva 2007; Tholl 2015).

For instance, monoterpenes and sesquiterpenes are key components of plant defense, serving to deter herbivores and pathogens while facilitating interspecies communication (Unsicker et al. 2009; Heil and Karban 2010; Huang et al. 2012). Growing evidence suggests they can also induce immune responses in neighboring plants through airborne signaling (Blande et al. 2014; Riedlmeier et al. 2017; Frank et al. 2021). In addition to their biological functions, these compounds significantly impact atmospheric chemistry and ecosystem dynamics, influencing aerosol formation and oxidative processes (Laothawornkitkul et al. 2009; Peñuelas and Staudt 2010).

Despite extensive research on terpenoids in plant defense, the role of isoprene ‐ the smallest and most abundant terpenoid ‐ remains debated. While isoprene is well known for protecting plants from abiotic stress, particularly high temperatures and oxidative damage (Velikova et al. 2011; Vanzo et al. 2016; Sharkey and Monson 2017), its function in biotic stress responses is less understood. Its thermo‐protective effects have been attributed to the modulation of reactive oxygen species (ROS) (Vickers et al. 2009a; Velikova et al. 2012). Recent evidence has provided further clarification of isoprene's role in maintaining the stiffness of thylakoid membranes and the stability of photosystem II under heat (Pollastri et al. 2019; Velikova et al. 2011). Further investigations suggest that isoprene indirectly influences stress protection by altering gene expression, protein abundance, and internal signaling pathways (Vanzo et al. 2016; Monson et al. 2021; Rosenkranz et al. 2021). Moreover, externally perceived isoprene has been shown to induce immunity in neighboring plants by modulating defense pathways and upregulating stress‐responsive genes (Harvey and Sharkey 2016; Frank et al. 2021).

Terpenoids play a key role in plant immunity through interactions with salicylic acid (SA) and jasmonic acid (JA) signaling pathways (Pieterse et al. 2012; Erb and Reymond 2019). SA is primarily associated with resistance against biotrophic pathogens, whereas JA is involved in defenses against necrotrophic pathogens and herbivores (Glazebrook 2005; Koornneef and Pieterse 2008; Betsuyaku et al. 2018). Studies on monoterpenes and sesquiterpenes indicate that specific compounds enhance plant resistance by interacting with these phytohormone pathways. For example, the sesquiterpene β‐caryophyllene enhances resistance to bacterial pathogens in *Arabidopsis thaliana* via JA signaling (Frank et al. 2021), while monoterpenes such as α‐pinene and β‐pinene, as well as isoprene, induce defense mechanisms dependent on SA biosynthesis and signaling (Riedlmeier et al. 2017; Frank et al. 2021; Pérez‐Pérez et al. 2024). Recent findings further suggest that monoterpenes function as airborne signals in plant‐to‐plant communication (Wenig et al. 2019). While the signaling roles of α‐pinene, β‐pinene, and β‐caryophyllene in plant defense are well‐characterized, the molecular mechanisms underlying isoprene‐mediated immunity and its interactions with other terpenoid‐induced defenses remain unclear.

A major challenge in BVOC research is translating laboratory findings to field conditions, where environmental variability ‐ such as fluctuating temperatures, light intensities, and biological interactions ‐ can strongly influence BVOC emission rates and plant responses (Loreto and Schnitzler 2010; Niinemets et al. 2013). In addition, the presence of background volatiles or the chemical degradation of emitted volatiles can obscure plant‐to‐plant communication in field conditions (Masui et al. 2022). For instance, terpenoids react quickly with ozone and OH radicals to form secondary oxidized volatiles (Hallquist et al. 2009). However, the study showed that HIPV‐mediated the responses in the receiver plants‐ including monoterpenes, are adjusted under ozone stress, but the defence benefits remain intact (Yu et al. 2022). Although recent studies have advanced our understanding of isoprene's role in plant defense against biotic stress, few have bridged the gap between controlled experiments and natural environments (Baldwin et al. 2006; Behnke et al. 2012).

Transgenic isoprene‐enhanced silver birch (*Betula pendula* Roth) (Bertić et al. 2023) presents a unique model for addressing these challenges. Silver birch, an ecologically and economically important tree species, primarily emits monoterpenes and sesquiterpenes but little isoprene (Hakola et al. 2001; Vuorinen et al. 2005). It plays a crucial role in boreal and temperate forest ecosystems, influencing nutrient cycling, biodiversity, and atmospheric chemistry (Hynynen et al. 2010; Ashburner et al. 2013). Transgenic silver birch lines with modified isoprene emission profiles provide an ideal system to investigate the effects of altered BVOC emissions on plant‐environment interactions and neighboring plants in natural settings (Ibrahim et al. 2010; Bertić et al. 2023).

This study aimed to determine whether plant‐released isoprene enhances the immunity of neighboring plants under natural field conditions. We used transgenic silver birch lines with varying isoprene emission levels (Bertić et al. 2023) and *Arabidopsis thaliana* as receiver plants, assessing their immune response by measuring suppression of *Pseudomonas syringae* growth. In addition to wild‐type *Arabidopsis*, we included isogenic knock‐out lines for *legume lectin‐like protein 1* (*llp1*) and *jasmonate resistance 1* (*jar1*), as these genes mediate monoterpene‐ (LLP1) and sesquiterpene‐induced (JAR1) immunity, respectively (Breitenbach et al. 2014; Wenig et al. 2019; Frank et al. 2021).

By conducting a 2‐year field experiment using transgenic birch trees and *Arabidopsis* mutants, this study provides new insights into the role of isoprene in plant immunity under natural conditions. We show that increasing isoprene emissions from silver birch enhance the immunity of neighboring plants against pathogens in the presence of other volatiles, providing new evidence for the role of isoprene in plant‐to‐plant communication and highlighting its potential in modulating the plant immunity in forest ecosystems.

## 2 Materials and Methods

### 2.1 Plant and Microbial Materials and Growth Conditions

**Figure 1:**
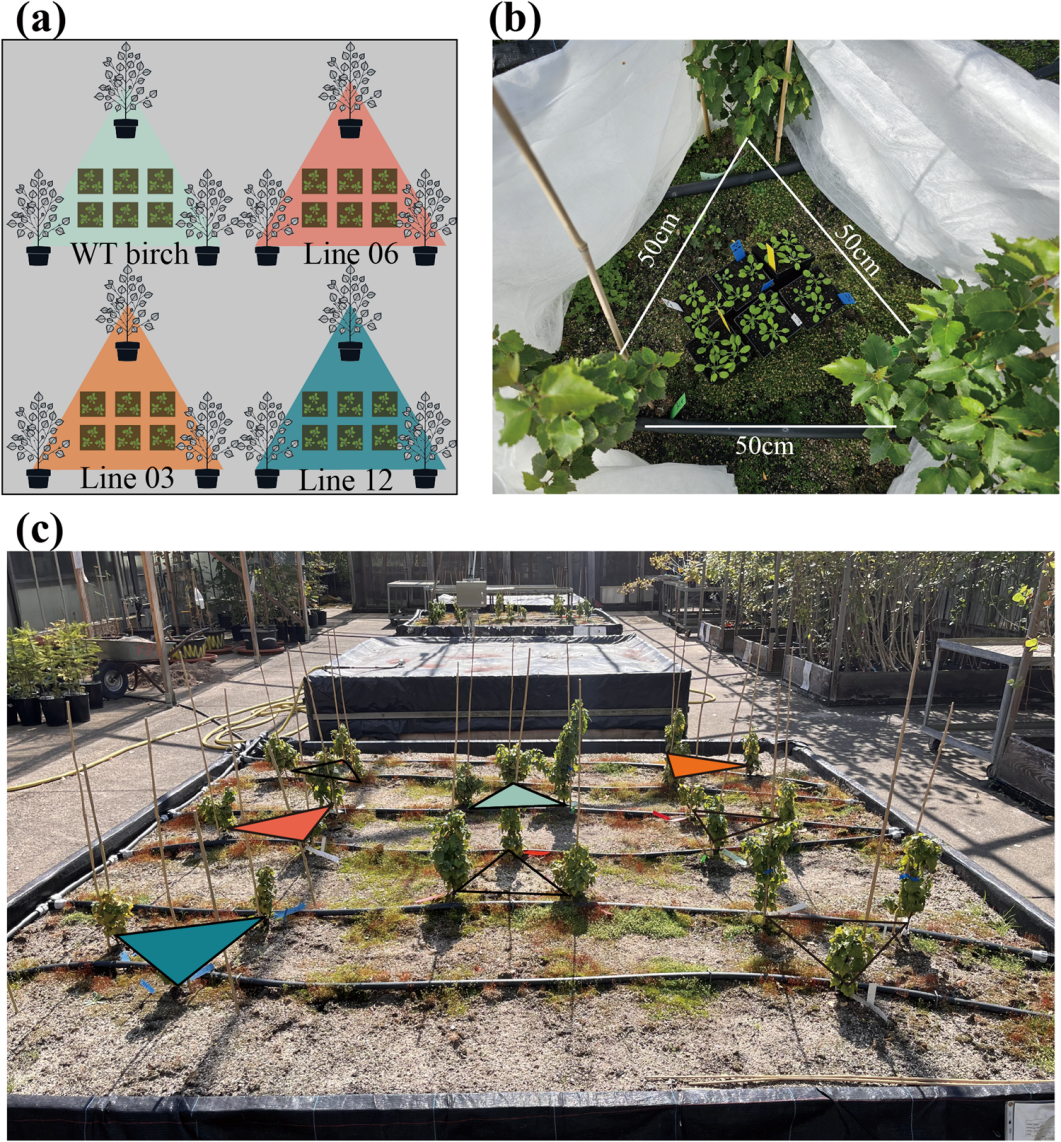
Experimental design for studying isoprene emission effects from transgenic silver birch on *Arabidopsis thaliana*. (a) Schematic representation of silver birch (*Betula pendula*) planting arrangement showing wild‐type (WT) and transgenic lines (06, 03, and 12) in triangular formations. (b) Magnified view of the central experimental area within a birch triangle, showing *Arabidopsis thaliana* plants (Col‐0 wild‐type, *llp1*, and *jar1* mutants) positioned for VOC exposure. (c) Field implementation of the experimental setup in an outdoor open‐air greenhouse showing the spatial arrangement of birch triangles with colored markers indicating different transgenic lines. [Color figure can be viewed at wileyonlinelibrary.com]

In the experiment, three genetically modified isoprene‐overexpressing lines of silver birch (lines 03, 06, and 12) clones were used alongside wild‐type silver birch (*Betula pendula* Roth). The transformation and cultivation procedures of these transgenic plants have been described previously (Bertić et al. 2023). In autumn 2021, the 4‐year‐old birch plants were transferred from the phytochambers to an outdoor, caged area of the Department of Forest Botany and Tree Physiology at the University of Göttingen, Germany. The experimental setup used to investigate the effect of isoprene emissions from transgenic silver birch on neighboring plants is illustrated in Figure 1. The young trees were grown in triangles of the same genotype at 50 cm distance in large soil‐filled boxes (Figure 1b,c). The plants over‐wintered outdoors and were used in subsequent years for the experiments.

In addition, wild‐type *Arabidopsis thaliana* (ecotype Columbia‐0 (Col‐0)) and the isogenic *Arabidopsis* KO mutant lines legume lectin‐like protein 1 (*llp1*) and jasmonate resistant 1 (*jar1*) were used. *Arabidopsis* plants were grown in climate‐controlled chambers in a soil mixture containing a 1:5 ratio of coarse sand to normal soil. Growth conditions were maintained at a temperature of 20/16°C and a relative humidity of 65/80% (day/night); light irradiation was provided at 100 μmol photons m^−2^ s^−1^ photosynthetic photon flux density (PPFD) with a 10‐h photoperiod. All *Arabidopsis* plants used in the experiments were 4.5 weeks old. *Arabidopsis* plants were not pre‐conditioned to outdoor conditions before the experiments.

The bacterial strain used in the infection assays was *Pseudomonas syringae* pv. *tomato* (Pst) DC3000. Bacteria were grown on NYGA medium (0.5% bactoproteose peptone, 0.3% yeast extract, 2% (v:v) glycerol, 1.8% agar, pH 7.0) supplemented with 50 μg·mL^−1^ rifampicin and 50 μg·mL^−1^ kanamycin at 28°C. For inoculation, freshly grown bacteria were suspended in 10 mM MgCl₂ and adjusted to OD₆₀₀ = 0.0002 (approximately 10⁵ colony forming units (CFU) mL^−1^). This inoculum concentration was chosen specifically to create a mild infection, enabling enhanced resistance to be detected sensitively without overwhelming the plant's defenses. This is consistent with previous studies on VOC‐mediated priming (Wenig et al. 2019). Two upper leaves of *Arabidopsis* plants were infiltrated with this bacterial suspension using a needleless syringe 84 h after the start of the volatile treatment. Three days after inoculation, *in planta* bacterial titers were determined as follows: three leaf discs per sample were shaken in 10 mM MgCl₂ with 0.01% (v:v) Silwet, serially diluted, plated on NYGA medium and incubated for 2 d at 28°C before colony counting and calculation of *in planta* bacterial titers (Wenig et al. 2019).

### 2.2 Exposure of *Arabidopsis* to Birch VOCs

To study the effect of isoprene emission on neighboring *Arabidopsis* receiver plants, the experiment was repeated three times independently as biological replicates. For each birch line, two triangular arrangements were established, with six pots of *Arabidopsis* plants (two pots per line: wild type, *llp1*, and *jar1*; three plants per pot) randomly placed in the center area of each triangle (Figure 1a,c). In 2023, an additional ambient air control group was included. In this group, *Arabidopsis* plants were placed in a soil box in the greenhouse at a sufficient distance from the experimental birch trees to avoid any effects. The *Arabidopsis* plants were arranged in the same manner as the VOC‐exposed group, including WT, *llp1* and *jar1* lines. Another, Grey poplar (*Populus* × *canescens*), a natural high‐isoprene emitter (Behnke et al. 2012), was included as a positive control using the same experimental setup as the birch treatments. During sampling, leaf material from the three *Arabidopsis* plants within a pot was pooled to form a single sample, consistent with methods described by Frank et al. (2021). To minimize wind disturbance, each triangular setup was enclosed with sticks and non‐woven fabric during the 84‐h exposure period (Figure 1b).

### 2.3 VOC Collection

To collect VOCs, the upper part, a terminal shoot (approximately 15–20 cm long) of each birch tree was covered with a transparent, disposable 0.5 L plastic coffee cup, which was gathered around the stem and secured tightly with Teflon tape to create an airtight enclosure. The PFA tubes were inserted through an inlet port on the bottom of the cup. Volatile sampling was performed using two MTS‐32 autosamplers (Markes International Ltd, Bridgend, UK) equipped with 2‐meter long 0.3 mm PFA tubes attached to each sampling port to enable air sample collection from different locations. The samples were adsorbed by GC glass tubes (Gerstel, Mülheim an der Ruhr, Germany) filled with 60 mg Tenax TA 60/80 and 60 mg Carbopack X 40/60 (both from Sigma‐Aldrich, St. Louis, USA). The sample flow rate was 250 ml min^−1^, maintained for 30 min. Six biological replicates per tree line were sampled. Empty cups sealed by Teflon bags placed on the soil box were sampled as the ambient air group. Before sampling, each tube was spiked with 2 µL of δ−2‐carene in methanol (429.7 pmol µL^−1^) as an internal standard. After sampling, the GC tubes were stored hermetically sealed at 4°C in a refrigerator for subsequent GC‐MS analysis.

### 2.4 VOC Analyses

The samples were analyzed as previously (Ghirardo et al. 2020) using thermo‐desorption gas chromatography‐mass spectrometry (TD‐GC‐MS; TD by Gerstel; GC type: 7890 A, MS type: 5975 C, both from Agilent Technologies, Palo Alto, CA, USA) and the following modified GC temperature ramp: initial temperature of 40°C increased at 10°C min^−1^ to 130°C, hold for 5 min, increased to 175°C at 80°C min‐1, then to 200°C at 2°C min^−1^, to 220°C at 4°C min^−1^, and finally to 300°C at 100°C min^−1^ and hold for 5 min. Quantitative and qualitative VOC analyses were performed using Agilent GC‐MS Enhanced ChemStation, version E.02.00.493 (Agilent, St. Clara, USA) as previously (Ghirardo et al. 2020), and compounds were annotated by spectral matching using the NIST20 and Wiley (v8) mass spectra libraries and by comparing the calculated non‐isothermal Kovats retention indices with those found in the literature (NIST Chemistry, WebBook SRD 69, webbook. nist. gov). Only compounds with a match quality score (ChemStation) greater than 75 were selected for further analysis. Compounds with scores of 75 or below were excluded. Compounds were quantified using calibration curves based on pure standards, and emission rates were calculated based on leaf area and enclosure time (Ghirardo et al. 2011, 2020).

### 2.5 Statistical Analysis

Orthogonal Partial Least Squares Discriminant Analysis (OPLS‐DA) was used to characterize the terpenoid profiles of the different silver birch lines. The pre‐processing of the raw GC‐MS data consisted of log10 transformation, mean centering and Pareto scaling. Before OPLS‐DA, principal component analysis (PCA) was performed to identify potential outliers. Samples that fell outside the 95% confidence Hotelling's T2 ellipse of the PCA were treated as outliers and excluded from further analysis. Subsequently, the volatile emission rates in the data matrix (21 × 38) were set as independent variables (X), while the categorical variables describing the different silver birch lines were set as dependent variables (Y) in the OPLS‐DA model. The aim of this analysis was to identify the main terpenoids characteristic of the different silver birch lines. The predictive performance of the model was assessed by the regression sum of squares (R2Y), the prediction sum of squares (Q2Y), the root mean square error of estimation (RMSEE) and the RMSEcv (root mean square error of cross‐validation). Models were tested for significance (*p* < 0.05) using the cross‐validated ANOVA procedure (Eriksson et al. 2008; Eriksson et al. 2013). OPLS‐DA score plots were plotted to illustrate the differentiation of birch lines based on their terpenoid emission profiles. All multivariate analyses were performed with the software package SIMCA‐P v.13.0.3.0 (Umetrics, Umea, Sweden).

Cell forming unit (CFU) data were first assessed for normality using the Shapiro‐Wilk test (*p* > 0.05). One‐way analysis of variance (ANOVA) was then conducted to evaluate differences among experimental groups, followed by Tukey's honestly significant difference (HSD) test for post‐hoc pairwise comparisons. For datasets with unequal group sizes, the Tukey‐Kramer method was applied. For terpenoid emission rates that did not meet the assumption of normality, the Kruskal‐Wallis‐test was used, followed by Dunn's test for post‐hoc comparisons. For bacterial growth analysis, percentage inhibition was calculated relative to appropriate within‐year controls (WT birch in 2022; ambient air in 2023) rather than using absolute CFU values for cross‐year comparisons, as baseline pathogen loads differed between years. Absolute CFU data are reported in Supplementary Table S2. All statistical analyses were performed using R software.

## 3 Results

### 3.1 Terpenoid Emission Profiles of Transgenic Silver Birch Lines

**Figure 2:**
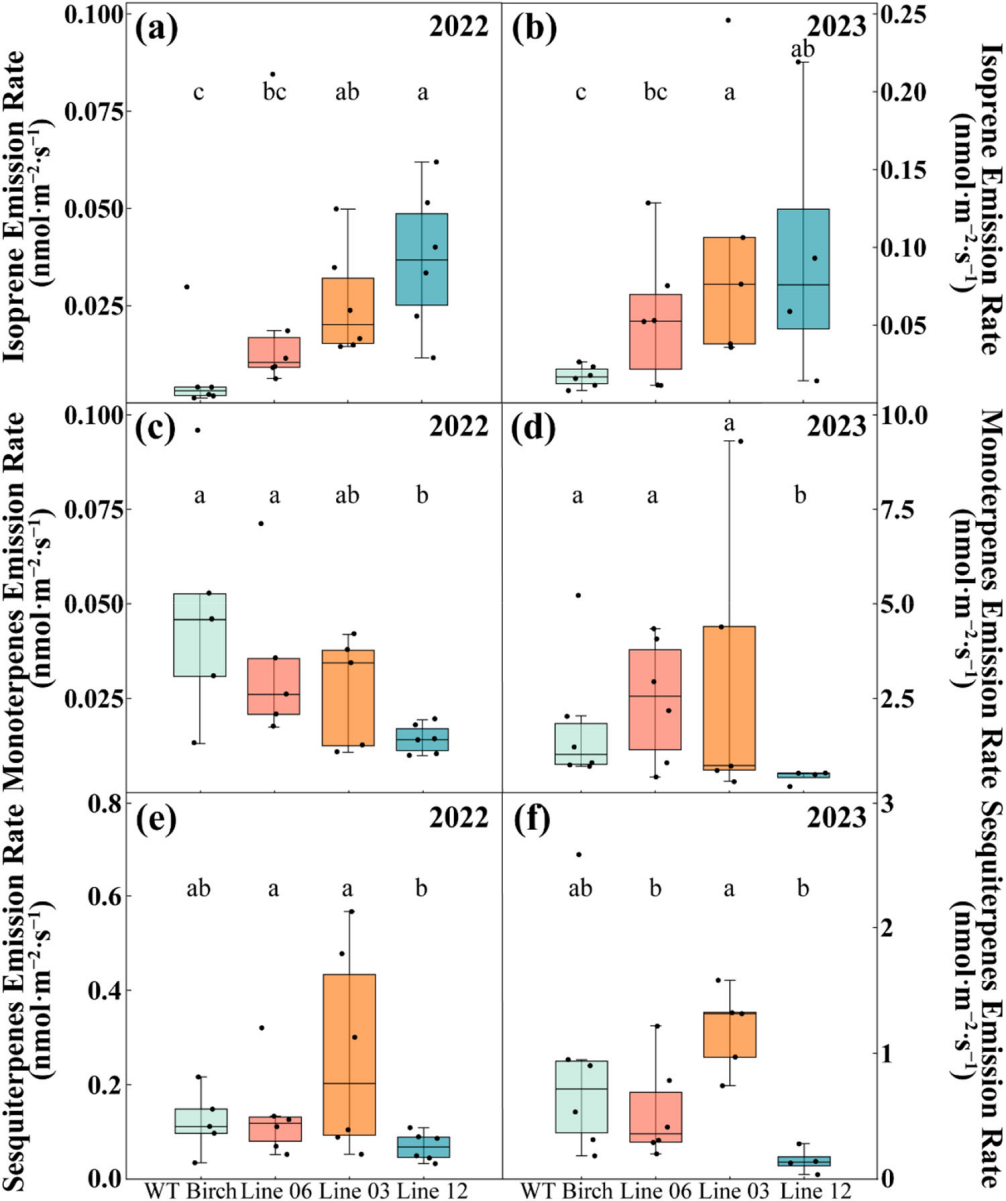
Volatile terpenoid emission rates from wild‐type and transgenic silver birch lines. Emission rates of (a, b) isoprene, (c, d) monoterpenes, and (e, f) sesquiterpenes measured from wild‐type (WT) *Betula pendula* and transgenic lines (06, 03, and 12) during growing seasons of 2022 (a, c, e) and 2023 (b, d, f). Box plots show median (horizontal line), interquartile range (box: 25th‐75th percentiles), and 10th‐90th percentiles (whiskers); *n* ≥ 4 biological replicates. Different letters indicate significant differences between lines (*p* < 0.05, Kruskal‐Wallis test with Dunn's post‐hoc comparison). [Color figure can be viewed at wileyonlinelibrary.com]

Transgenic modifications significantly altered terpenoid emissions across different compound classes, with patterns remaining consistent over two growing seasons (Figure 2). Line effects were clearly observed for isoprene emissions. In 2022, birch line 12 emitted the highest levels, followed by line 03 with intermediate levels (*p* < 0.05), while wild‐type (WT) birch exhibited the lowest emissions (Figure 2a). In line with these observations, the ratio of monoterpene to isoprene carbon numbers in 2022 was significantly lower in line 12 than in WT birch (see Supplementary Figure S1a). This suggests that carbon allocation favors isoprene in this line, which emits high levels of isoprene. A similar trend was observed in 2023, with both lines 03 and 12 showing significantly higher emissions than WT, while line 06 had intermediate levels (Figure 2b; *H₂* = 10.231, *df* = 3, *p* < 0.05, *η²* = 0.425).

Monoterpene emission patterns of birches appeared inversely related to isoprene emission in 2022, WT birch and line 06 were the highest emitters (Figure 2c), while line 12 showed significantly lower emissions (*p* < 0.05). However, in 2023 this inverse pattern was not obvious, monoterpenes emission increased for all lines (Figure 2d), but line 12 still showed reduced emissions compared to other lines (Figure 2d; *H₂* = 7.198, *df* = 3, *p* < 0.1). In 2023, sesquiterpene emissions exhibited significant differences (*H₂* = 8.703, *df* = 3, *p* < 0.05, *η²* = 0.34), with line 03 emitting notably higher levels (1.157 ± 0.146 nmol m⁻²·s⁻¹) compared to lines 06 and 12 (0.517 ± 0.152 and 0.236 ± 0.105 nmol m⁻²·s⁻¹, respectively). Although the relative emission patterns between the birch genotypes remained consistent across both years, absolute emission rates were higher in 2023, likely to correspond to higher sampling temperatures (Supplementary Figure S2). Additionally, the older birch trees in 2023 may have contributed to their higher emission capacity, compared to 2022. Beyond the major terpenoid classes emitted, sporadic trace emissions of methyl salicylate (MeSA) were observed, although below reliable quantification levels (see Supplementary Figure 3).

### 3.2 Isoprene From *Betula pendula* Affects *Arabidopsis thaliana* Immune Responses

To investigate the potential effects of different terpenoid emissions on plant immunity, we examined whether the differential emissions from transgenic birch lines induce plant immunity and inhibit bacterial growth in WT *Arabidopsis*.

**Figure 3:**
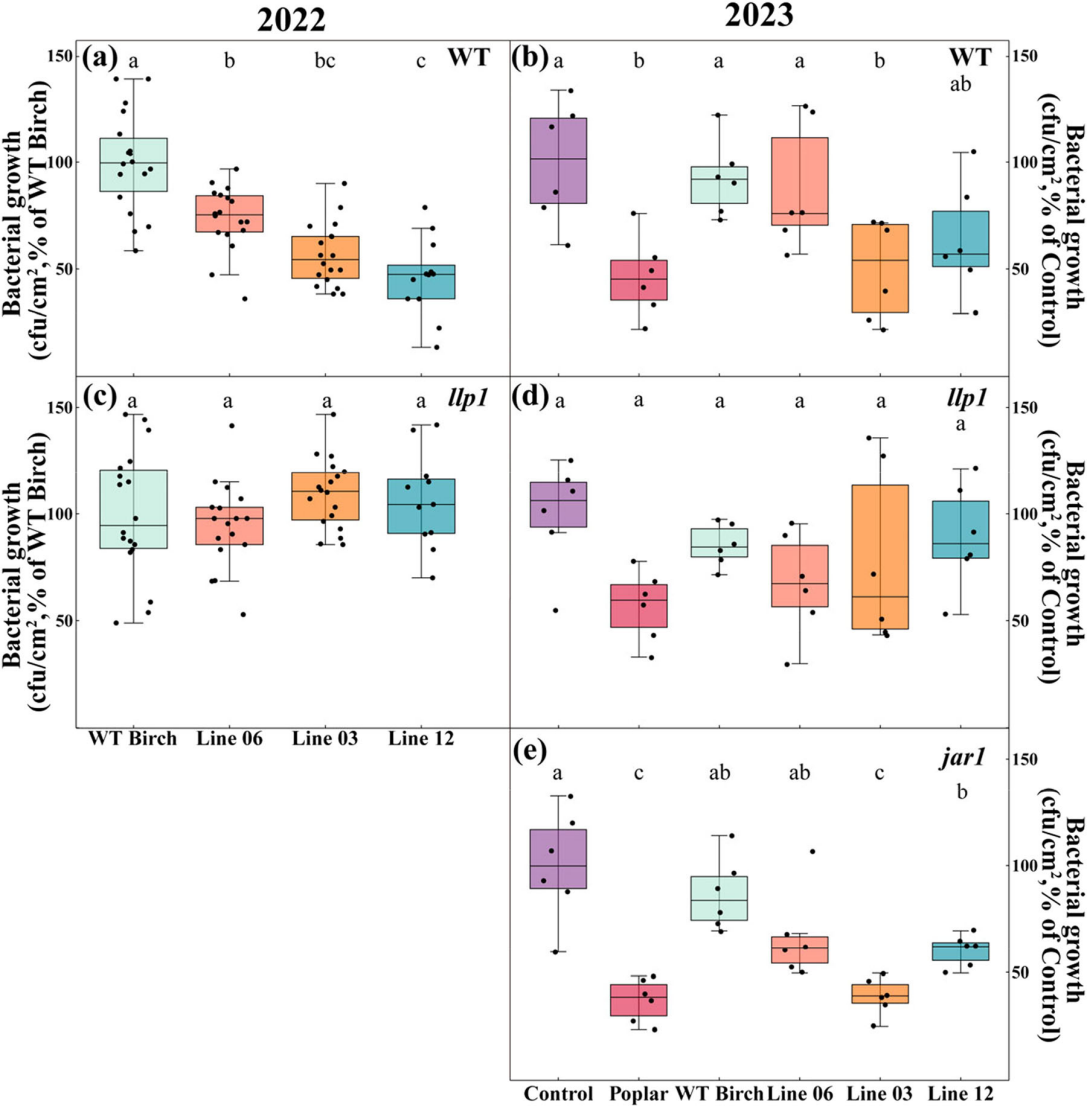
Impact of silver birch volatile emissions on bacterial growth in wild‐type and mutant *Arabidopsis thaliana* (*llp1* and *jar1*). Bacterial growth quantification in (a, b) wild‐type (WT), (c, d) *llp1* mutant, and (e) *jar1* mutant *A. thaliana plants* exposed to VOCs from control conditions (no plant emissions), grey poplar (*Populus × canescens*, positive control), wild‐type *Betula pendula*, and three transgenic birch lines (06, 03, and 12) during 2022 (WT and *llp1*) and 2023 (WT, *llp1*, and *jar1*). Bacterial growth is expressed as percentage relative to plants exposed to WT birch emissions (2022) or ambient air/control treatment (2023). Poplar was included as a positive control in 2023 for isoprene‐mediated effects on bacterial growth. Box plots display median (center line), interquartile range (box boundaries: 25th–75th percentiles), and range excluding outliers (whiskers); individual points represent biological replicates (*n* ≥ 5 per treatment). Different letters indicate significant differences between treatments (*p* < 0.05, one‐way ANOVA followed by Tukey‐Kramer post hoc test). [Color figure can be viewed at wileyonlinelibrary.com]

Over the 2 years, exposure to volatiles from transgenic birch lines induced varying degrees of bacterial growth inhibition in WT *Arabidopsis*. Line 03 consistently showed significant effects (*p* < 0.05); in 2023, line 03 performed similarly to the positive control and showed significant bacterial growth inhibition compared to the control and WT birch, while the effect of line 12 was stronger in 2022 compared to 2023 (Figure 3a,b). However, line 06, which emitted lower isoprene levels (higher than WT birch but lower than line 03 and 12), significantly inhibited bacterial growth only in 2022, but showed no significant effect in 2023.

In 2023, we included ambient air as an additional control. WT *Arabidopsis* exposed to ambient air showed bacterial growth levels similar to low‐emitting treatment groups (WT birch and line 06), but significantly higher than those exposed to high isoprene‐emitting lines (line 03 and 12). Line 12 consistently emitted significantly lower monoterpenes than WT birch, while its sesquiterpene emissions showed lower but not significant difference compared to WT (Figure 2c–f). However, line 12's high isoprene emissions were still associated with strong bacterial growth inhibition in receiver plants, suggesting that isoprene plays a specific role in this response.

**Figure 4:**
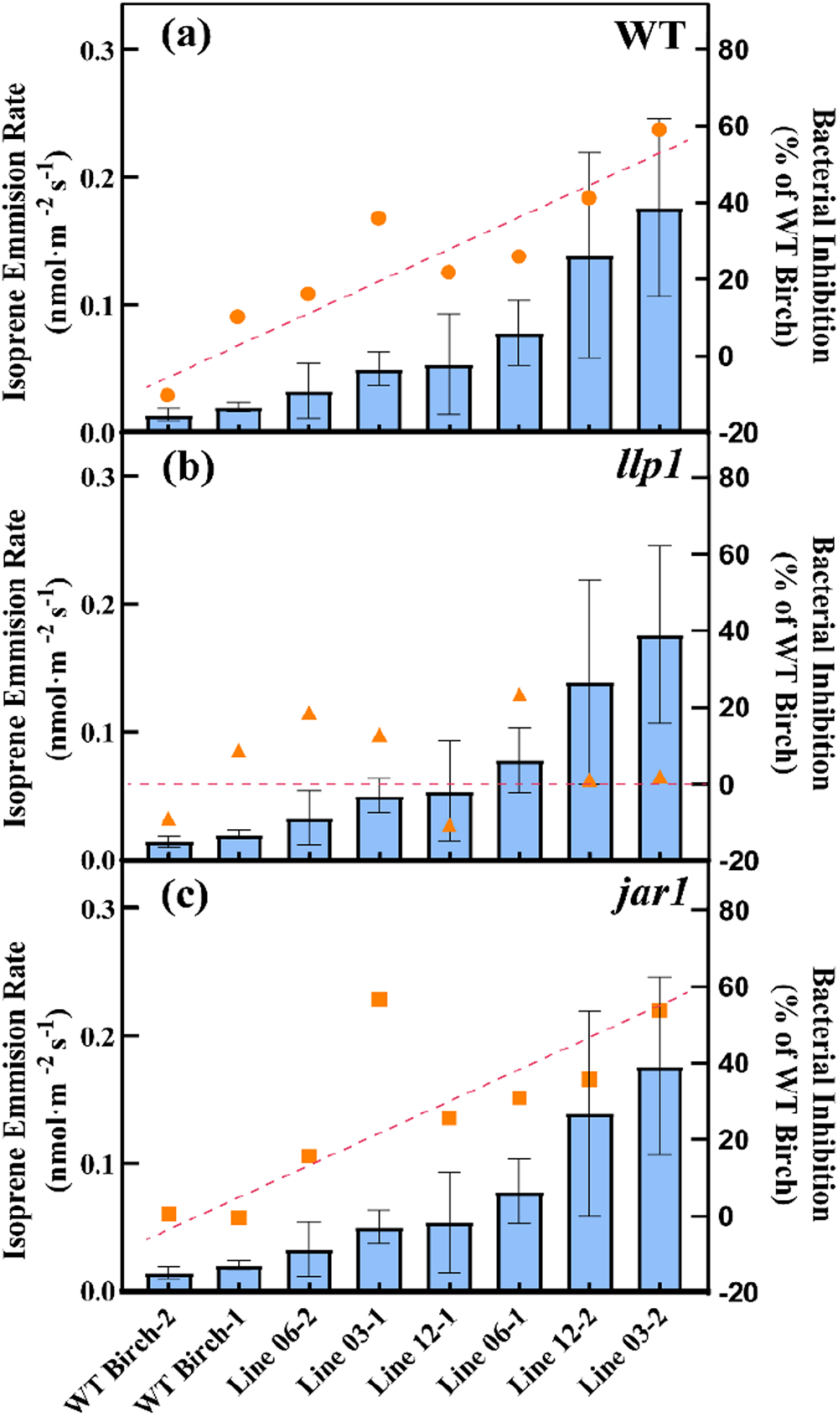
Correlation between isoprene emission rates and bacterial inhibition for each triangle in wild‐type (WT) and mutant birch lines with defective defense signaling pathways. Isoprene emission rates (blue bars, mean ± SEM) and bacterial inhibition (orange markers, % of WT Birch) are presented for (a) wild‐type (WT), (b) *llp1* mutant (defective in monoterpene‐mediated salicylic acid defense signaling), and (c) *jar1* mutant (impaired in sesquiterpene‐mediated jasmonic acid defense signaling) birch lines. Red dashed lines indicate the linear regression between emission rates and bacterial inhibition. Statistical analysis revealed a significant positive correlation in WT *Arabidopsis* (*R²* = 0.7881, *p* = 0.0032). In contrast, no significant correlation was observed for *llp1* (*R²* = 0.0094, *p* = 0.8195) or *jar1* (*R²* = 0.4908, *p* = 0.0529) mutant lines. Numbers following line designations represent different emission triangles. [Color figure can be viewed at wileyonlinelibrary.com]

**Figure 5:**
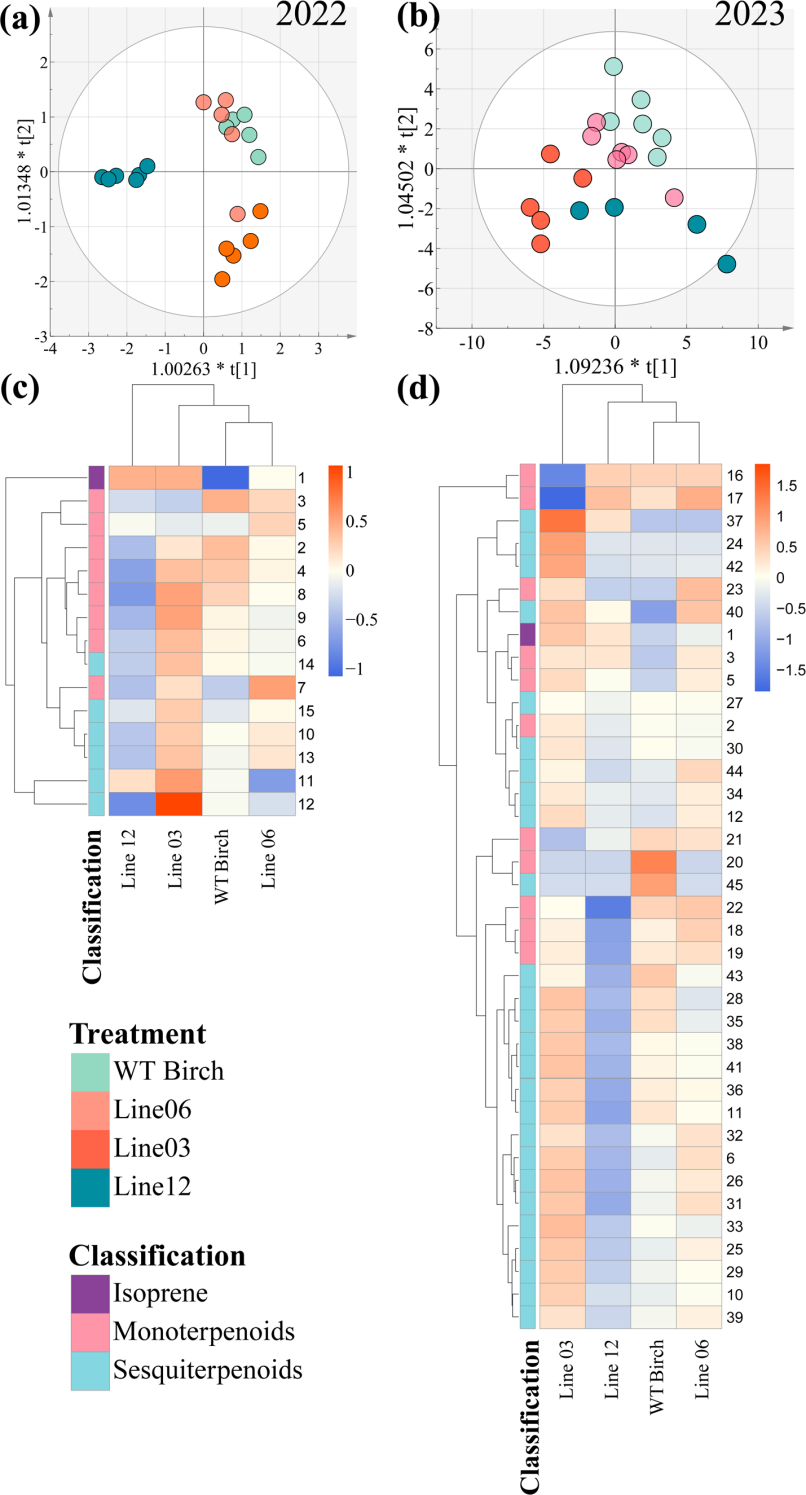
Multivariate and cluster analysis of terpenoid emissions from wild‐type and transgenic silver birch lines in 2022 and 2023. (a‐b) Orthogonal partial least squares discriminant analysis (OPLS‐DA) score plots of terpenoid emissions from wild‐type (WT) birch and three transgenic lines (06, 03, and 12) for the experiments performed in 2022 (a) and 2023 (b). Each point represents an individual sample. OPLS‐DA model fitness: (a) *R* ^*2*^ *X (cum)* = 0.875, *R* ^*2*^ *Y(cum)* = 0.86, *Q* ^*2*^ *Y(cum)* = 0.752, using predictive and two orthogonal components. CV‐ANOVA, *p* = 0.0002. (b) *R* ^*2*^ *X*(cum) = 0.752, *R* ^*2*^ *Y*(cum) = 0.898, *Q* ^*2*^ *Y*(cum) = 0.626, using three predictive and two orthogonal components. CV‐ANOVA, *p* = 0.003. (c‐d) Clustered heatmaps showing the relative abundance (means of *n* ≥ 4 biological replicates) of individual terpenoids (rows) across different birch lines (columns) for 2022 (c) and 2023 (d). Dendrograms on the left show the hierarchical clustering of terpenoids based on their emission patterns, using Euclidean distance and complete linkage. Numbers on the right of heatmap (c, d) correspond to individual terpenoid compounds (Supplementary Table S1). [Color figure can be viewed at wileyonlinelibrary.com]

To quantitatively analyze the relationship between isoprene emission and bacterial growth inhibition, we performed correlation analysis using the actual isoprene emission rates and bacterial suppression levels measured for each experimental group in 2023 (Figure 4a). Given the varying emission patterns among individual birch trees (Figures 2 and 5), we calculated the average isoprene emission rate for each experimental group surrounding the *Arabidopsis* receivers. Statistical analyses revealed significant differences (*p* < 0.05) among the experimental groups and a strong positive correlation (*R²* = 0.7881, *p* = 0.0032) between isoprene emission levels and bacterial growth inhibition in the receiver plants. Notably, no significant relationship was found between monoterpene or sesquiterpene emissions and bacterial growth inhibition on any genotype (Supplementary Figure S4). This supports isoprene as the primary volatile signal inducing plant immunity in neighboring plants, consistent with our laboratory assay using the pure compound before the field study (Supplementary Figure S5). We also tested correlations between bacterial inhibition and individual mono‐ and sesquiterpenoids in 2023. None of these correlations, however, were significant (see Supplementary Table S3).

### 3.3 Impact of Birch VOCs on Bacterial Growth in *Arabidopsis* Mutants

To further explore the effects of silver birch VOCs on plant immunity in *Arabidopsis* receivers, we examined bacterial growth responses in *Arabidopsis* mutants with defects in distinct defense signaling pathways: *jar1* (impaired in jasmonate signaling) and *llp1* (deficient in monoterpene‐induced systemic acquired resistance).

In bacterial growth assays with *llp1* mutants, no significant differences were observed between birch genotypes in either 2022 or 2023 (Figure 3c,d). Correlation analyses revealed no significant relationships between terpenoid emissions and bacterial growth inhibition in these mutants (*p* > 0.05; Figure 4b and Supplementary Figure S4). This complete loss of VOC‐induced resistance in *llp1* mutants suggests that this signaling pathway plays a critical role in perceiving birch volatiles.

In contrast, *jar1* mutants exhibited genotype‐specific responses to birch VOCs in 2023 (Figure 3e). High isoprene‐emitting lines 03 and 12 induced significant bacterial growth suppression in *jar1* mutants compared to ambient air controls and WT birch exposure (*p* < 0.05). Correlation analysis revealed a moderate positive relationship between isoprene emission rates and bacterial growth inhibition in *jar1* mutants (*R²* = 0.4908, *p* = 0.0529; Figure 4c), while neither monoterpenes (*R²* = 0.0500, *p* = 0.5947) nor sesquiterpenes (*R²* = 0.0349, *p* = 0.6577) showed significant correlations (Supplementary Figures S4e and S4f). These results suggest that jasmonate signaling contributes to, but is not essential for, isoprene‐induced immunity. The roles of other signaling pathways and the genetic requirements for responses to other volatile compounds still need further investigation.

To further validate the role of isoprene in inducing plant immunity, we included grey poplar (*Populus × canescens*), a well‐known strong emitter of isoprene, as a positive control in 2023. Exposure to poplar emissions induced strong bacterial growth inhibition in WT *Arabidopsis*, similar to that observed with high‐isoprene birch lines. However, this effect was completely abolished in *llp1* mutants but preserved in *jar1* mutants (see Figure 3), confirming the LLP1‐dependent, but JAR1‐independent pathway observed with transgenic birch emissions.

## 4 Discussion

Our experimental design, using transgenic silver birch trees with enhanced isoprene emissions and *Arabidopsis* plants as receivers, demonstrates the role of isoprene in mediating plant immunity under natural conditions in the presence of other volatiles. The consistent response patterns observed over 2 years, despite environmental variations, indicate that isoprene plays a role in plant‐to‐plant communication. While higher temperatures in 2023 were associated with increased isoprene emissions (see Supplementary Figures S1 and S2), the relationship between relative isoprene emissions and bacterial resistance remained similar. This suggests that the defense mechanism is reliable across different environmental conditions. Recent studies have further highlighted the critical role of temperature and light intensity in modulating VOC emissions and plant responses under field conditions (Nagalingam et al. 2023; Ma et al. 2023). This is particularly true for isoprene, whose biosynthesis and emission respond quickly to changes in temperature and light, with emission rates closely tracking environmental fluctuations over short periods (Sharkey et al. 1999; Lehning et al. 2001).

Notably, although the genotypic differences in isoprene emission rates we reported are statistically significant, they are small in absolute terms, compared to strong isoprene emitters such as poplar. Emission rates and plant biomass affect air concentration, which has implications for signal distance in plant‐to‐plant communication. We also recognize that low emission rates may have limited the strength of induced responses in pathogen resistance. However, the fact that significant resistance trends are consistent with these small emission differences suggests that isoprene is biologically relevant in the proximity of the isoprene source. It is noteworthy that higher isoprene emissions in 2023 did not lead to stronger inhibition of bacterial growth compared with 2022. This nonlinear relationship is consistent with Frank et al. (2021), who showed that emissions from 1 to 2 poplar leaves effectively induced resistance, whereas emissions from four leaves diminished the effect. Such an “optimal concentration window” has also been demonstrated for monoterpenes (Riedlmeier et al. 2017) and sesquiterpenes, that is, β‐caryophyllene (Frank et al. 2021), and may reflect threshold effects, receptor saturation, or modulation by other volatiles and environmental factors that shape both VOC emissions and plant physiology. Direct comparison of absolute emission values across years is further complicated by differing environmental conditions (Supplementary Figure S2), which influence not only the emitter's output but also the receiver's physiological state and perception threshold. Ultimately, the immune response depends on both the signal and the receiver's context‐dependent capacity to perceive and respond.

The constant VOC emission patterns observed in the birch mutants over 2 years prompted us to explore the metabolic relationships within terpenoid biosynthesis. The inverse relationship between isoprene and monoterpene emission rates we observed (especially in 2022) in consistent with previously reported trade‐offs in isoprene biosynthesis (Harrison et al. 2013), suggesting a metabolic trade‐off in the plastidic methyl‐ d‐erythritol phosphate (MEP) pathway. This crosstalk likely reflects multiple regulatory mechanisms, including competition for common precursors (IPP and DMAPP), differential enzyme kinetics, and transcriptional regulation of biosynthetic genes in the MEP pathway (Ghirardo et al. 2014; Volke et al. 2019). The complex interactions between introduced genes and the plant's native genome can result to unexpected phenotypes or metabolic shifts (Huang et al. 2018; Fan et al. 2020). While increased isoprene emissions are typically linked to decreased monoterpene and sesquiterpene emissions, birch line 03 displayed high emissions of both terpenoid groups, indicating complex metabolic adaptations that warrant further investigation (Ali et al. 2018; Hu et al. 2023). Despite the consistent VOC emission pattern, in the VOC profiles year‐to‐year variations were observed. In 2022, the different transgenic birch lines exhibited clear differences (see Figure 5), which are consistent with the genotypic differences reported in the 4‐year‐old birch trees before the establishment of the field trial (Bertić et al. 2023). In 2023, however, a decrease in differences between the lines was observed, potentially due to the trees maturing and different environmental factors. The higher overall VOC emissions in 2023 (a result of plant development and the ambient climate) may have masked earlier genotype comparisons. These results suggest that the birch metabolism is influenced by both growth and environmental factors, probably causing the reduced differences in VOC emission rates between lines in the second year.

Although birch and *Arabidopsis* rarely co‐occur in nature, our findings demonstrate that constitutively emitted plant volatiles such as isoprene can prime the immune response of neighboring plants under natural environmental conditions. The observation of this interspecific effect between species lacking a close ecological relationship suggests that such VOC‐mediated interactions could be even more significant and finely tuned in natural, co‐evolved plant communities. Consequently, VOC‐mediated plant‐to‐plant signaling mechanisms may also be significant in ecosystems, for example in arctic and subarctic tundra ecosystems, where a high number of species emit isoprene and terpenoids (Li et al. 2023), and plants often grow in close proximity. While overall vegetation density varies, dense plant patches can occur (Ghirardo et al. 2020), and VOCs are known to be actively emitted and to play ecological roles (Ghirardo et al. 2020; Rieksta et al. 2023). Similarly, in tropical ecosystems, hyper‐dominant species in the Amazon, such as *Inga* and *Protium*, demonstrate isoprene's role in enhancing resilience to environmental stress (Taylor et al. 2018; Jardine et al. 2020). In these ecosystems, isoprene emissions are associated with thermotolerance and the mitigation of oxidative stress, providing an adaptive advantage to species that emit it under specific environmental conditions. These findings suggest that isoprene could influence community composition by affecting survival and plant performance, but further research is needed to understand how it directly affects broader community dynamics in different ecosystems. Moreover, isoprene emission has been shown to deter insect herbivory by priming plant defense pathways, highlighting its role in mediating biotic as well as abiotic stress responses (Sahu et al. 2025).

The pathway‐specific nature of this communication system is further confirmed by the differential responses in mutant *Arabidopsis* lines. The varying responses observed in WT and mutant *Arabidopsis* lines (*llp1* and *jar1*) suggest that isoprene's effects on plant immunity involve the LLP1 gene, a component of the SA mediated systemic acquired resistance (SAR) signaling pathway (Wenig et al. 2019; Frank et al. 2021). Similarly, monoterpene‐induced immunity depends on functional LLP1, indicating overlapping responses to isoprene and monoterpenes through the SAR regulatory network (Riedlmeier et al. 2017; Wenig et al. 2019; Frank et al. 2021). However, our findings regarding LLP1 differ from the laboratory study of Frank et al. (2021), likely due to the complex volatile mixtures and environmental variables present in field conditions. While MeSA is known to mediate plant communication and systemic acquired resistance (Shulaev et al. 1997; Baldwin et al. 2006), effective signaling typically requires much higher concentrations than those detected in our birch samples. In contrast, the preserved responsiveness of *jar1* mutants suggests that JA‐dependent defenses may play a minor role in isoprene‐induced immunity. While isoprene emissions were significantly correlated with bacterial growth inhibition (Figure 4), neither monoterpene nor sesquiterpene emissions showed similar correlations in our study (Supplementary Figure S4, Supplementary Table S3). These results highlight the complexity of plant immune responses to VOCs and suggest that plants may use multiple pathways to enhance immunity (Junker et al. 2018).

Crucially, the dependence of the induced resistance on the plant genetic component LLP1, strongly suggests that the immune response is mediated by the plant itself, rather than resulting from a direct antimicrobial effect of isoprene on *Pseudomonas syringae*. If isoprene were to act directly on the pathogen, the *Arabidopsis llp1* mutant would be expected to exhibit outcomes similar to those of the WT, rendering the *llp1* genotype irrelevant. This interpretation is consistent with studies demonstrating that although isoprene possesses antimicrobial properties, effective concentrations (240–720 ppm) are orders of magnitude higher than those found in the natural environment (Yu et al. 2022). In our study, pathogen growth inhibition in vivo serves as a proxy for immune strength, yet how plants perceive volatile terpenoids, especially isoprene, remains unresolved. Current evidence indicates multimodal perception: protein‐mediated routes for some stress VOCs alongside indirect effects on membranes/redox state (Loreto and D'Auria 2022). A specific protein interactor has been implicated for a sesquiterpene: TOPLESS/TOPLESS‐like corepressors in β‐caryophyllene responses (Nagashima et al. 2019), linking volatile cues to gene regulation. In silico docking suggests several putative plant OBP‐like proteins bind monoterpenes, whereas isoprene shows minimal affinity (Giordano et al. 2021), implying it likely bypasses OBP‐mediated pathways. A leading alternative is indirect perception via rapid *in planta* oxidation products, methyl vinyl ketone (MVK) and methacrolein (MACR), which can trigger defense‐associated transcription; plants mitigate their phytotoxicity by metabolism/conjugation (Tani et al. 2020) and by alkenal/one oxidoreductases that reduce MVK to methyl ethyl ketone (MEK) (Canaval et al. 2020). Together, these findings support a model in which monoterpenes and some sesquiterpenes act through specific protein interactors, whereas isoprene responses are more plausibly mediated by its oxidation products and are modulated by receiver physiology (e.g., ROS, temperature, SAR components).

In line with this framework, future work should quantify downstream signalling readouts, both SAR‐associated markers (e.g., canonical defence genes) and electrophile/ROS‐responsive and detoxification markers (e.g., pathways handling MVK/MACR), to clarify how an isoprene‐derived signal is processed. Our *llp1* results point to SAR involvement, yet the nonlinear concentration dependence of isoprene signalling warrants targeted dose–response tests. As shown by Frank et al. (2021), moderate emissions from one to two poplar leaves induced resistance, whereas higher emissions were ineffective, indicating an optimal concentration window rather than a monotonic dose–response. Defining the threshold for effective signalling, and how it shifts with receiver state (e.g., temperature, ROS status) and with the levels of isoprene oxidation products, will be critical to resolve when and how the signal is perceived.

Moreover, laboratory experiments with *Arabidopsis* overexpressing the isoprene synthase gene from *Eucalyptus* have shown that isoprene production alters signaling networks related to specific phytohormones, such as gibberellic acid, and stress tolerance (Zuo et al. 2019). This underscores the need for future studies, especially under natural conditions, to include additional defense‐related mutants beyond *llp1* and *jar1*, particularly those involved in MAPK cascades and calcium signaling, to gain a deeper understanding of how isoprene integrates into broader immune networks (Zander et al. 2020; Liu et al. 2020; Johnson et al. 2023). Furthermore, multi‐omics approaches, such as RNA‐Seq, untargeted metabolomics, and post‐translational modification (PTM) proteomics, could provide insights into the molecular mechanisms underlying VOC‐mediated interactions and help identify key regulatory hubs (Shang and Huang 2019; Zhao et al. 2023; Chen et al. 2024). Recent studies on ectomycorrhizal fungal‐plant interactions have shown the potential of these integrative approaches to unravel plant VOC signaling networks (Plett et al. 2024).

Our study demonstrates that isoprene can enhance plant immunity over short distances ( < 50 cm) under natural conditions, adding to its known role in protecting photosystems against thermal stress (Sharkey et al. 1995; Behnke et al. 2007) and oxidative damage (Loreto and Velikova 2001; Vickers et al. 2009b). However, these plant‐level benefits must be balanced against isoprene's atmospheric chemistry effects, particularly its contribution to ozone formation in NOx‐rich environments (Atkinson and Arey 2003) and secondary organic aerosol formation (Zhao et al. 2023; Bryant et al. 2023). Based on our findings, we propose a context‐dependent approach for managing isoprene‐emitting species. In agricultural settings and natural ecosystems distant from urban areas, the benefits of isoprene emission for plant health and community resilience may outweigh its atmospheric impacts. In urban and suburban regions with prevalent NOx pollution, however, priority should be given to low‐isoprene‐emitting species to minimize air quality impacts (Rosenkranz et al. 2015). This spatial optimization strategy allows us to leverage the beneficial effects of isoprene for plant health while minimizing its negative atmospheric consequences in pollution‐sensitive areas.

Although we have revealed the ecological value of isoprene as a volatile signaling molecule, the dose‐effect relationship, the threshold for regulation by environmental factors, and the chemical nature of signal perception remain to be clarified. An attractive hypothesis is that the real trigger for immunity may be the rapid oxidation of isoprene within leaves to generate methyl vinyl ketone (MVK) or methacrolein (MACR) (Jardine et al. 2012), metabolites that have been shown to induce defense responses and amplify MeSA and sesquiterpene emissions in poplar (Canaval et al. 2020). Future research is needed to accurately quantify the concentration thresholds of isoprene and its oxidation products under natural conditions and elucidate the localization of isoprene in coupled plant‐microbe‐atmosphere networks by integrating data from the transcriptome, metabolome and microbial communities (including isoprene oxidizers; e.g. Gibson et al. 2021) to understand how variables such as temperature, light intensity, and vapor pressure deficit synergistically shape this response. By expanding the experimental framework of this study, we will be able to more comprehensively assess the ecological functions of isoprene and its oxidation products in coupled plant‐atmosphere networks and provide operational science for vegetation management and air quality regulation.

## Supporting information


**Figure S1:** Carbon number ratios of terpenoid emissions from wild‐type and transgenic silver birch (*Betula pendula*) lines in 2022 and 2023.
**Figure S2:** Environmental conditions during experimental periods in 2022 and 2023.
**Figure S3:** Methyl salicylate (MeSA) emission rates from wild‐type and transgenic silver birch lines.
**Figure S4:** Monoterpene and sesquiterpene emission rates in birch lines and their correlation with bacterial growth inhibition in *Arabidopsis thaliana* mutants.
**Figure S5:** Laboratory pre‐test with pure pinene and isoprene reduced leaf bacterial growth.
**Table S1:** List of isoprene, monoterpenoids and sesquiterpenoids identified in birch lines with their retention times and Kovats index.
**Table S2:** Bacterial titers in *Arabidopsis thaliana* plants exposed to volatile emissions from wild‐type and transgenic silver birch lines and poplar (*Populus* x *canescens*).
**Table S3:** Correlation analysis of individual monoterpene and sesquiterpene emission rates with pathogen inhibition across different birch lines in 2023.

## Data Availability

The data that support the findings of this study are available on request from the corresponding author. The data are not publicly available due to privacy or ethical restrictions. The data that support the findings of this study are available on request from the corresponding author. The data is not publicly available due to privacy or ethical restrictions.
