# Supplementary material for: Volatile‐Mediated Plant Defense Networks: Field Evidence for Isoprene as a Short‐Distance Immune Signal
Source: Plant Cell Environ. 2025 Sep 1;48(12):8616–29. doi: 10.1111/pce.70153 (PMC12586919; doi:10.1111/pce.70153)
Supplement: Supplementary file 1 — Figure S1: Carbon number ratios of terpenoid emissions from wild‐type and transgenic silver birch (Betula pendula) lines in 2022 and 2023. Figure S2: Environmental conditions during experimental periods in 2022 and 2023. Figure S3: Methyl salicylate (MeSA) emission rates from wild‐type and transgenic silver birch lines. Figure S4: Monoterpene and sesquiterpene emission rates in birch lines and their correlation with bacterial growth inhibition in Arabidopsis thaliana mutants. Figure S5: Laboratory pre‐test with pure pinene and isoprene reduced leaf bacterial growth. Table S1: List of isoprene, monoterpenoids and sesquiterpenoids identified in birch lines with their retention times and Kovats index. Table S2: Bacterial titers in Arabidopsis thaliana plants exposed to volatile emissions from wild‐type and transgenic silver birch lines and poplar (Populus x canescens). Table S3: Correlation analysis of individual monoterpene and sesquiterpene emission rates with pathogen inhibition across different birch lines in 2023. [file PCE-48-8616-s001.docx]

**Supporting Information for**

Volatile-Mediated Plant Defense Networks: Field Evidence for Isoprene as a Short-Distance Immune Signal

Peiyuan Zhu^1^, Baris Weber^1^, Maaria Rosenkranz^1,2^, Andrea Polle^3,4^, Andrea Ghirardo^1^,

Jan Muhr^3,4^, A. Corina Vlot^5^, Jörg-Peter Schnitzler^1*^

*Corresponding author: Jörg-Peter Schnitzler

**Email:** [joergpeter.schnitzler@helmholtz-munich.de](mailto:joergpeter.schnitzler@helmholtz-munich.de)

**Supplemental Figures and Tables**

Supplemental Figure S1


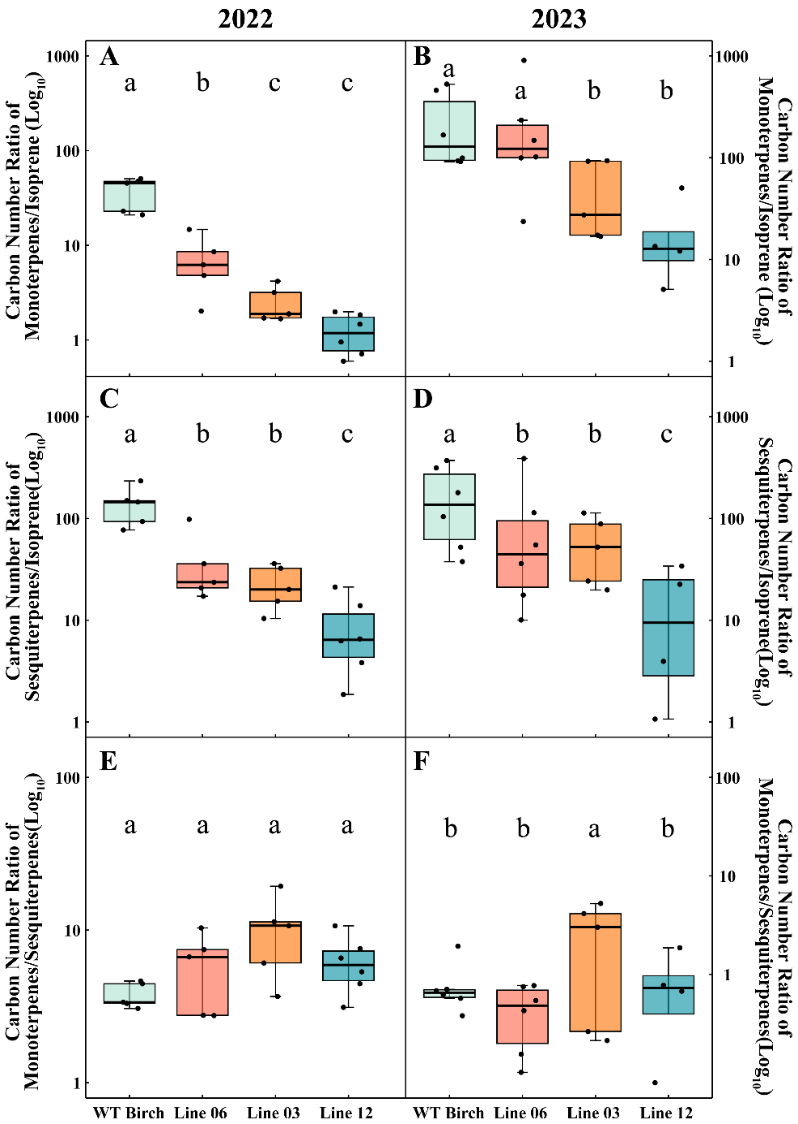


**Figure S1: Carbon number ratios of terpenoid emissions from wild-type and transgenic silver birch (*Betula pendula*) lines in 2022 and 2023.** Carbon number ratios of different terpenoid classes emitted by wild-type (WT) birch and three transgenic lines (06, 03, and 12) over two consecutive years. (a, b) Monoterpenes to isoprene ratio. (c, d) Sesquiterpenes to isoprene ratio. (e, f) Sesquiterpenes to monoterpenes ratio. Note the difference in scale. The boxes represent the interquartile range (IQR), with the median indicated by the horizontal line. Whiskers extend to 1.5 times the IQR, and data points beyond this range are shown as individual dots (*n* ≥ 4). Different letters indicate significant differences between treatments (*P* < 0.05, one-way ANOVA).

Supplemental Figure S2

**
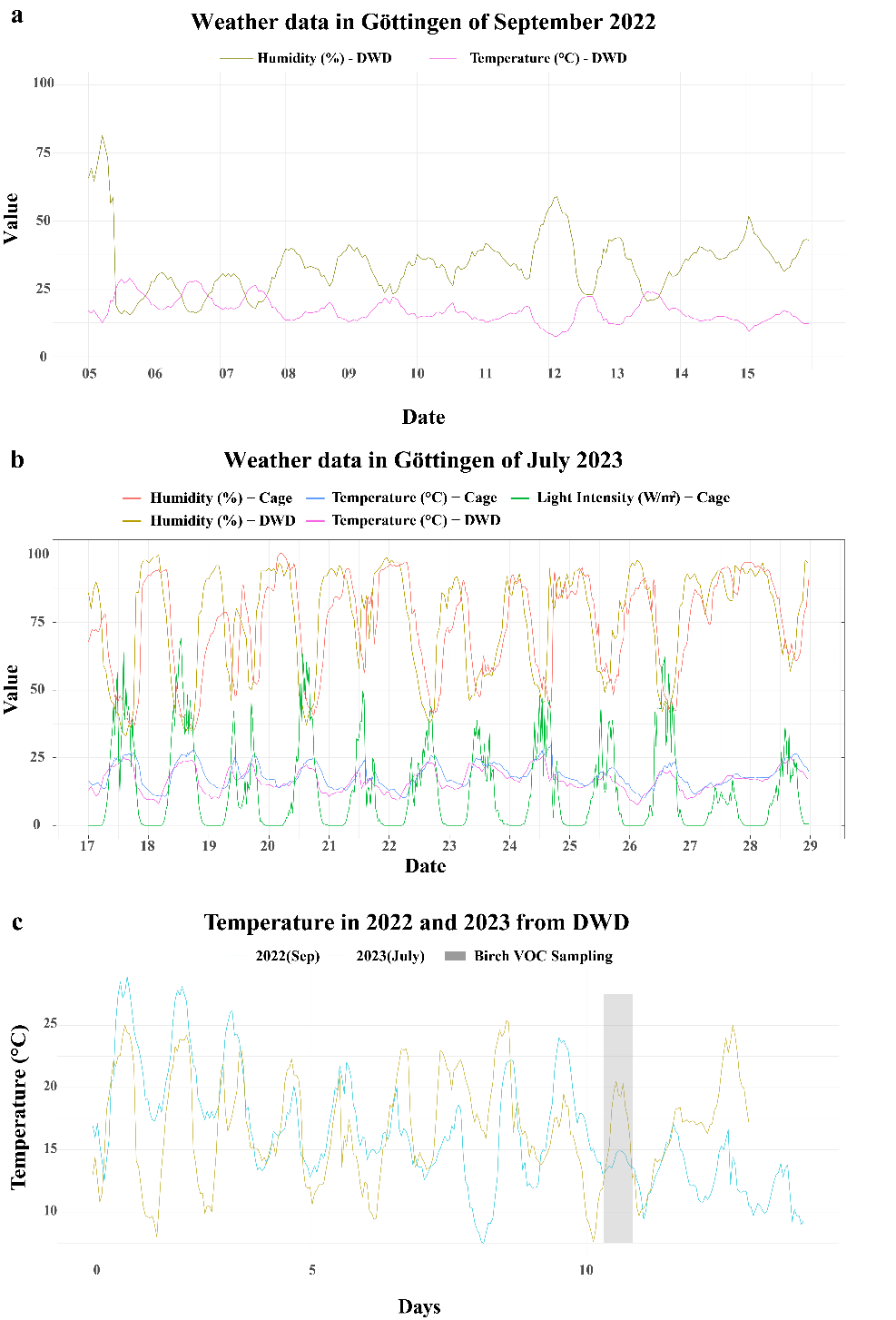
**

**Figure S2. Environmental conditions during experimental periods in 2022 and 2023.** (a) Temperature (°C, pink line) and relative humidity (%, yellow/green line) measured at the DWD (Deutscher Wetterdienst, German Weather Forecast Service) weather station in Göttingen from September 5–15, 2022. (b) Daily temperature (°C, blue lines), relative humidity (%, red/yellow lines), and light intensity (W/m^2^, green line) recorded in the experimental cage and at the DWD weather station from July 17–29, 2023. (c) Comparison of temperature trends between September 2022 (light blue line) and July 2023 (orange line) from DWD data over a 12-day period. The grey shaded area marks the timing of birch VOC sampling. The temperature data illustrates temporal variation and differences in thermal conditions between the two experimental periods.Supplemental Figure S3

**
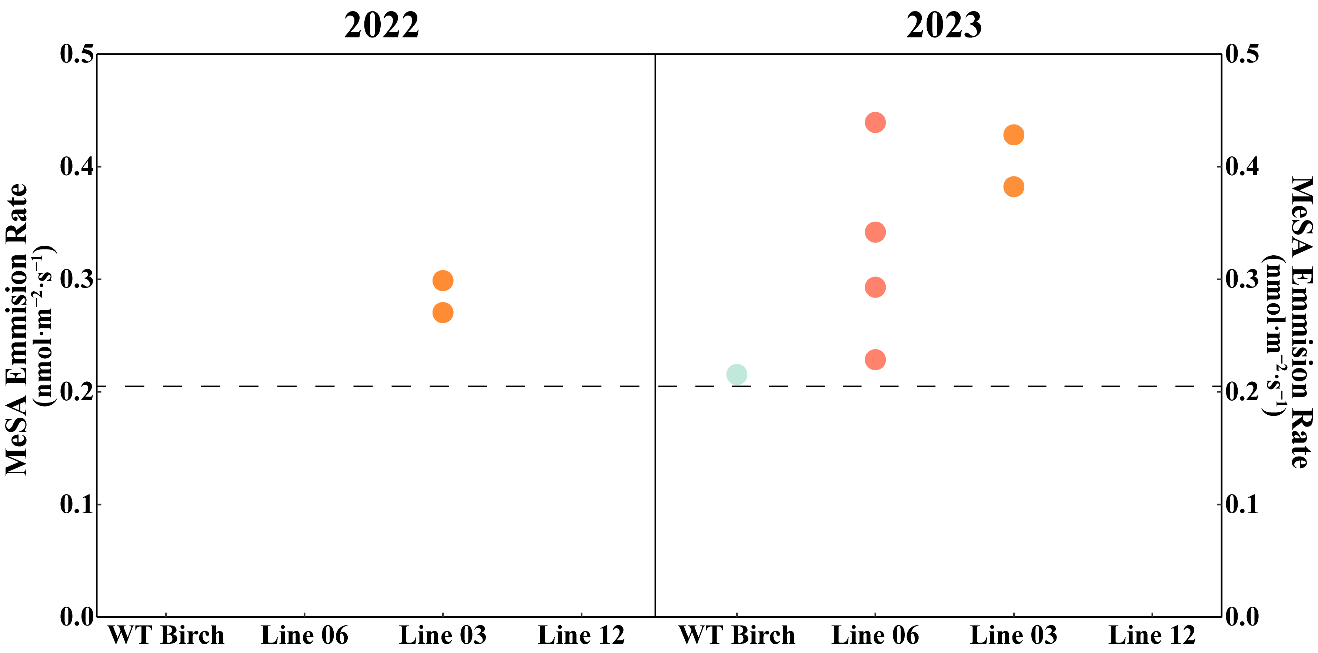
**

**Figure S3. Methyl salicylate (MeSA) emission rates from wild-type and transgenic silver birch lines.** MeSA emission rates measured from wild-type (WT) *Betula pendula* and transgenic lines (06, 03, and 12) during the growing seasons of 2022 and 2023. The dashed horizontal line indicates the limit of quantification (LOQ), determined as the lowest calibration standard producing stable chromatographic peaks. Only individual measurements above LOQ are shown. Sample sizes: 2022 - *n* = 6 per line; 2023 - WT (*n* = 6), Line 06 (*n* = 6), Line 03 (*n* = 5), Line 12 (*n* = 4). In 2022, quantifiable MeSA emissions were detected only in Line 03 (2/6 replicates). In 2023, quantifiable emissions were detected in WT (1/6), Line 06 (4/6), and Line 03 (2/5). Line 12 showed no quantifiable MeSA emissions in either year. Individual data points are shown. No statistical analysis was performed due to insufficient sample sizes.

Supplemental Figure S4


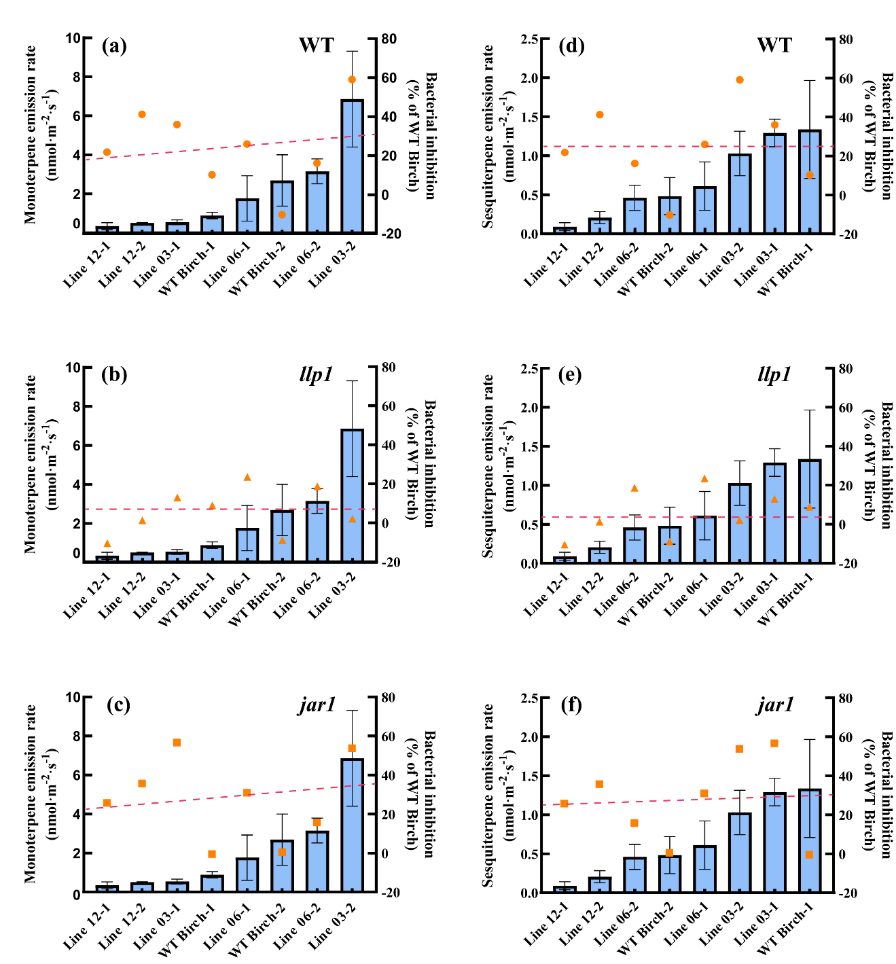


**Figure S4. Monoterpene and sesquiterpene emission rates in birch lines and their correlation with bacterial growth inhibition in *Arabidopsis* *thaliana* mutants.** Emission rates of (a-c) monoterpenes and (d-f) sesquiterpenes were measured in different birch lines triangles and tested against (a, d) wild-type (WT) *Arabidopsis*, (b, e) *llp1* mutant (salicylic acid signaling-deficient), and (c, f) *jar1* mutant (jasmonic acid signaling-deficient). Blue bars represent VOC emission rates from birch lines (mean ± SEM, *n* = 3). Orange symbols indicate bacterial growth inhibition in *Arabidopsis* genotypes relative to WT control (%). Red dashed lines illustrate the trend between VOC emissions and antimicrobial activity. Correlation analysis showed no significant relationships for monoterpenes [monoterpenes: WT (*R²* = 0.1212, *P* = 0.3980), *llp1* (*R²* = 0.009380, *P* = 0.8195), *jar1* (*R²* = 0.04996, *P* = 0.5947)] and sesquiterpenes [WT (*R²* = 0.02893, *P* = 0.6872), *llp1* (*R²* = 0.1343, *P* = 0.3719), *jar1* (*R²* = 0.03493, *P* = 0.6577)]. The numbers following birch line designations represent different emission triangles.

Supplemental Figure S5

**
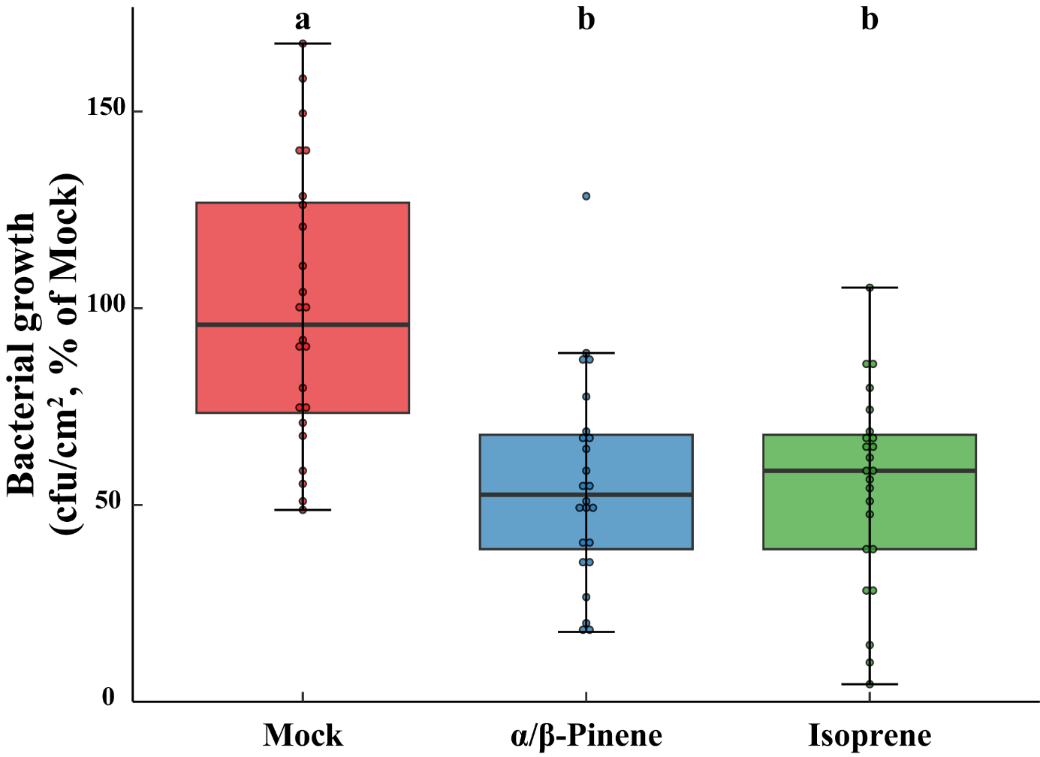
**

**Figure S5. Laboratory pre-test with pure pinene and isoprene reduced leaf bacterial growth.**

*Arabidopsis thaliana* plants were exposed in the desiccator to pure α/β-pinene (2.7 ppm) or isoprene (87.1 ppm); mock with hexane only. Leaf bacterial growth was quantified as cfu·cm⁻², expressed relative to the Mock treatment (100%) and log₁₀-transformed prior to analysis. Boxplots show medians (center lines), interquartile ranges (boxes), and 1.5×IQR whiskers; points are biological replicates (n ≥ 6). Different letters denote significant differences among treatments (one-way ANOVA with Tukey’s HSD, *P*< 0.05): both pinene and isoprene are lower than mock, and they do not differ from each other.

**Table S1. List of isoprene, monoterpenoids and sesquiterpenoids identified in birch lines with their retention times and Kovats index.**

| **Num** | **Compound** | **Retention Time** | **CAS Number** | **Kovats Index** | **Classification** |
| --- | --- | --- | --- | --- | --- |
| 1 | isoprene | 5.710 | 78-79-5 | 705 | Isoprene |
| 2 | α-pinene^*^ | 9.668 | 80-56-8 | 894 | Monoterpenes |
| 3 | D-limonene^*^ | 11.519 | 5989-27-5 | 982 | Monoterpenes |
| 4 | β-cyclocitral | 15.307 | 432-25-7 | 1164 | Monoterpenoids |
| 5 | (-)-car-3-en-2-one | 16.120 | 53585-45-8 | 1202 | Monoterpenoids |
| 6 | *trans*-α-bergamotene | 19.538 | 13474-59-4 | 1366 | Sesquiterpenes |
| 7 | 7-epi-sesquithujene | 18.554 | 159407-35-9 | 1319 | Sesquiterpenes |
| 8 | γ-elemene | 19.654 | 29873-99-2 | 1371 | Sesquiterpenes |
| 9 | valerena-4,7(11)-diene | 19.694 | 351222-66-7 | 1373 | Sesquiterpenes |
| 10 | valencene | 19.978 | 4630-07-3 | 1387 | Sesquiterpenes |
| 11 | α-farnesene | 20.775 | 502-61-4 | 1425 | Sesquiterpenes |
| 12 | β-selinene | 20.900 | 17066-67-0 | 1431 | Sesquiterpenes |
| 13 | unknown sesquiterpene 1 | 19.908 | - | 1383 | Sesquiterpenes |
| 14 | unknown sesquiterpene 2 | 20.949 | - | 1433 | Sesquiterpenes |
| 15 | unknown sesquiterpene 3 | 21.448 | - | 1457 | Sesquiterpenes |
| 16 | sabinene | 10.269 | 3387-41-5 | 923 | Monoterpenes |
| 17 | β-myrcene | 10.434 | 123-35-3 | 931 | Monoterpenes |
| 18 | *trans*-β-ocimene | 11.249 | 3779-61-1 | 970 | Monoterpenes |
| 19 | β-ocimene | 11.488 | 13877-91-3 | 981 | Monoterpenes |
| 20 | linalool | 12.709 | 78-70-6 | 1039 | Monoterpenoids |
| 21 | *(E)*-4,8-dimethylnona-1,3,7-triene | 12.994 | 19945-61-0 | 1053 | Monoterpenes |
| 22 | neo-allo-ocimene | 13.408 | 7216-56-0 | 1073 | Monoterpenes |
| 23 | allo-ocimene | 13.823 | 57396-75-5 | 1093 | Monoterpenes |
| 24 | α-cubebene | 17.889 | 17699-14-8 | 1287 | Sesquiterpenes |
| 25 | ylangene | 18.386 | 14912-44-8 | 1311 | Sesquiterpenes |
| 26 | (-)-β-bourbonene | 18.706 | 5208-58-2 | 1326 | Sesquiterpenes |
| 27 | α-santalene | 19.261 | 512-61-8 | 1353 | Sesquiterpenes |
| 28 | β-copaene | 19.374 | 18252-44-3 | 1358 | Sesquiterpenes |
| 29 | α-bergamotene | 19.496 | 18252-46-5 | 1364 | Sesquiterpenes |
| 30 | sesquisabinene | 19.622 | 58319-04-3 | 1370 | Sesquiterpenes |
| 31 | *cis*-β-farnesene | 19.686 | 28973-97-9 | 1373 | Sesquiterpenes |
| 32 | 6,9-guaiadene | 19.769 | 36577-33-0 | 1377 | Sesquiterpenes |
| 33 | β-santalene | 19.845 | 511-59-1 | 1380 | Sesquiterpenes |
| 34 | sesquisabinene B | 20.032 | 1431636-59-7 | 1389 | Sesquiterpenes |
| 35 | β-guaiene | 20.185 | 88-84-6 | 1397 | Sesquiterpenes |
| 36 | 1,3,6,10-dodecatetraene, 3,7,11-trimethyl-, *(Z,E)*- | 20.419 | 26560-14-5 | 1408 | Sesquiterpenes |
| 37 | β-bergamotene | 20.599 | 15438-94-5 | 1416 | Sesquiterpenes |
| 38 | germacrene D | 20.683 | 23986-74-5 | 1420 | Sesquiterpenes |
| 39 | β-bisabolene | 20.982 | 495-61-4 | 1435 | Sesquiterpenes |
| 40 | sesquicineole | 21.174 | 90131-02-5 | 1444 | Sesquiterpenoids |
| 41 | γ-cadinene | 21.307 | 39029-41-9 | 1450 | Sesquiterpenes |
| 42 | β-sesquiphellandrene | 21.365 | 20307-83-9 | 1453 | Sesquiterpenes |
| 43 | sesquirosefuran | 21.725 | 39007-93-7 | 1470 | Sesquiterpenoids |
| 44 | 7-epi-*trans*-sesquisabinene hydrate | 21.841 | 145512-84-1 | 1476 | Sesquiterpenoids |
| 45 | 7-epi-*cis*-sesquisabinene hydrate | 21.940 | 11028-42-5 | 1481 | Sesquiterpenoids |

*Compounds verified using authentic standards

**Table S2. Bacterial titers in *Arabidopsis thaliana* plants exposed to volatile emissions from wild-type and transgenic silver birch lines and poplar (*Populus* x *canescens*).**

| **Year** | **Birch Line**  **Treatment** | ***Arabidopsis* Genotype** | **Mean Titer**  **(×10⁵ CFU/cm^2^)** | **SE**  **(×10⁵ CFU/cm²)** | **n** | **Statistical Group*** |
| --- | --- | --- | --- | --- | --- | --- |
| 2022 | WT Birch | Wild type | 2.831 | 0.020 | 18 | a |
| 2022 | Line 06 | Wild type | 2.031 | 0.130 | 18 | b |
| 2022 | Line 03 | Wild type | 1.507 | 0.105 | 18 | bc |
| 2022 | Line 12 | Wild type | 1.147 | 0.178 | 12 | c |
| 2022 | WT Birch | *llp1* | 3.712 | 0.343 | 18 | a |
| 2022 | Line 06 | *llp1* | 3.627 | 0.324 | 18 | a |
| 2022 | Line 03 | *llp1* | 4.002 | 0.279 | 18 | a |
| 2022 | Line 12 | *llp1* | 3.944 | 0.289 | 12 | a |
| 2023 | Ambient air/Control | Wild type | 43.63 | 5.106 | 6 | a |
| 2023 | WT Birch | Wild type | 40.45 | 3.154 | 6 | a |
| 2023 | Line 06 | Wild type | 38.43 | 5.318 | 6 | a |
| 2023 | Line 03 | Wild type | 21.76 | 4.168 | 6 | b |
| 2023 | Line 12 | Wild type | 27.71 | 4.766 | 6 | ab |
| 2023 | Poplar | Wild type | 20.17 | 3.341 | 6 | b |
| 2023 | Ambient air/Control | *llp1* | 61.47 | 6.281 | 6 | a |
| 2023 | WT Birch | *llp1* | 52.44 | 2.476 | 6 | a |
| 2023 | Line 06 | *llp1* | 41.40 | 6.038 | 6 | a |
| 2023 | Line 03 | *llp1* | 48.51 | 10.57 | 6 | a |
| 2023 | Line 12 | *llp1* | 54.88 | 6.148 | 6 | a |
| 2023 | Poplar | *llp1* | 35.03 | 4.102 | 6 | a |
| 2023 | Ambient air/Control | *jar1* | 51.38 | 5.431 | 6 | a |
| 2023 | WT Birch | *jar1* | 44.59 | 3.523 | 6 | ab |
| 2023 | Line 06 | *jar1* | 34.18 | 4.359 | 6 | ab |
| 2023 | Line 03 | *jar1* | 19.96 | 1.829 | 6 | c |
| 2023 | Line 12 | *jar1* | 30.89 | 1.541 | 6 | b |
| 2023 | Poplar | *jar1* | 19.00 | 2.076 | 6 | c |

**Table S3. Correlation analysis of individual monoterpene and sesquiterpene emission rates with pathogen inhibition across different birch lines in 2023.**

| **Terpenoid Compound** | **Correlation Coefficient (Spearman's r) with Bacterial Inhibition** | ***P*-value** | **Significance  (*P* < 0.05)** |
| --- | --- | --- | --- |
| α-pinene | -0.1103 | 0.7949 | ns |
| D-limonene | 0.133 | 0.7535 | ns |
| sabinene | -0.138 | 0.7445 | ns |
| β-myrcene | 0.07581 | 0.8584 | ns |
| *trans*-β-ocimene | 0.3837 | 0.3481 | ns |
| β-ocimene | 0.1744 | 0.6795 | ns |
| linalool | -0.4675 | 0.2428 | ns |
| (*E*)-4,8-dimethylnona-1,3,7-triene | 0.5399 | 0.1672 | ns |
| neo-allo-ocimene | 0.2142 | 0.6106 | ns |
| allo-ocimene | 0.2227 | 0.5961 | ns |
| (-)-car-3-en-2-one | 0.3879 | 0.3424 | ns |
| α-cubebene | 0.1502 | 0.7226 | ns |
| ylangene | 0.4834 | 0.2249 | ns |
| *trans*-α-bergamotene | 0.4788 | 0.23 | ns |
| (-)-β-bourbonene | -0.08984 | 0.8325 | ns |
| α-santalene | -0.3792 | 0.3542 | ns |
| β-copaene | 0.3064 | 0.4604 | ns |
| α-bergamotene | 0.3826 | 0.3496 | ns |
| Sesquisabinene | 0.3386 | 0.412 | ns |
| *cis*-β-farnesene | 0.3405 | 0.4091 | ns |
| 6,9-guaiadene | 0.2489 | 0.5523 | ns |
| β-santalene | 0.08224 | 0.8465 | ns |
| valencene | 0.1859 | 0.6595 | ns |
| sesquisabinene B | 0.55 | 0.1579 | ns |
| β-guaiene | 0.4625 | 0.2486 | ns |
| 1,3,6,10-dodecatetraene, 3,7,11-trimethyl-, (*Z,E*)- | 0.341 | 0.4085 | ns |
| β-bergamotene | 0.6678 | 0.0703 | ns |
| germacrene D | 0.04547 | 0.9149 | ns |
| α-farnesene | 0.3303 | 0.4242 | ns |
| β-selinene | -0.1767 | 0.6754 | ns |
| β-bisabolene | -0.02805 | 0.9474 | ns |
| sesquicineole | 0.4838 | 0.2245 | ns |
| γ-cadinene | 0.3445 | 0.4034 | ns |
| β-sesquiphellandrene | 0.6989 | 0.0538 | ns |
| sesquirosefuran | 0.1624 | 0.7008 | ns |
| 7-epi-*trans*-sesquisabinene hydrate | 0.5466 | 0.161 | ns |
| 7-epi-*cis*-  sesquisabinene hydrate | -0.6557 | 0.0775 | ns |
